# Gastroesophageal Variceal Bleed Post Transcatheter Embolisation of the Left Gastric Artery

**DOI:** 10.7759/cureus.85233

**Published:** 2025-06-02

**Authors:** James M Ryley, Samad Abdul, Paul Ng

**Affiliations:** 1 Gastroenterology, University Hospitals Sussex, Brighton, GBR; 2 Gastroenterology, Frimley Health NHS Foundation Trust, London, GBR

**Keywords:** chronic pancreatitis (cp), gastric variceal bleeding, non cirrhotic portal hypertension, pseudoaneurysm, splenic vein thrombus

## Abstract

Chronic pancreatitis has several complications that can lead to upper gastrointestinal bleeding (UGIB). Patients with chronic pancreatitis presenting with evidence of UGIB require careful assessment and investigation. We report a case of a patient presenting with anaemia and general fatigue with a background of chronic pancreatitis. He had known non-cirrhotic portal hypertension secondary to a chronic splenic vein thrombus. The patient was found to have a left gastric artery pseudoaneurysm. Following embolisation, the patient had episodes of haematemesis, and oesophagogastroduodenoscopy (OGD) identified bleeding gastroesophageal varices (GOV). We hypothesise a mechanism of increased blood flow into the splenic venous system post-embolisation, leading to variceal rupture.

## Introduction

Chronic pancreatitis can result in complications such as splenic vein thrombosis and gastric varices, both of which predispose patients to upper gastrointestinal bleeding (UGIB) [1]. Vascular complications are secondary to several proposed mechanisms. Splenic vein thrombosis causes local inflammation, endothelial damage, and pseudocysts, causing local compression on the venous system. The formation of pseudoaneurysms is felt to be secondary to the degradation and inflammation of arteries surrounding the pancreas [2].

Endoscopy is typically the first line of investigation in patients who present with a suspected UGIB [3]. However, chronic pancreatitis may also give rise to other bleeding sources, such as pseudoaneurysms, necessitating angiography for a definitive diagnosis [4]. These cases present significant diagnostic and therapeutic challenges. We report a case of gastric variceal bleeding that occurred following the embolisation of the left gastric artery for the treatment of a pseudoaneurysm.

## Case presentation

A male in his 60s presented with generalised fatigue and had developed a feeling of dizziness and therefore presented to the emergency department. He had no symptoms of abdominal pain, malaena or haematemesis. Laboratory investigations revealed profound anaemia, with haemoglobin levels of 39 g/L. The patient had been investigated extensively with multiple oesophagogastroduodenoscopies (OGD) and colonoscopies, which had revealed only portal hypertensive gastropathy. Therefore, a computed tomography angiogram (CTA) was conducted to locate the source of anaemia. His medical history included atrial fibrillation managed with a left atrial appendage occlusion device, recurrent anaemia of unclear aetiology, chronic pancreatitis, splenic vein thrombosis, and type 2 diabetes. Liver imaging was normal, and his most recent fibroscan measured 5.8 kPa.

**Figure 1:**
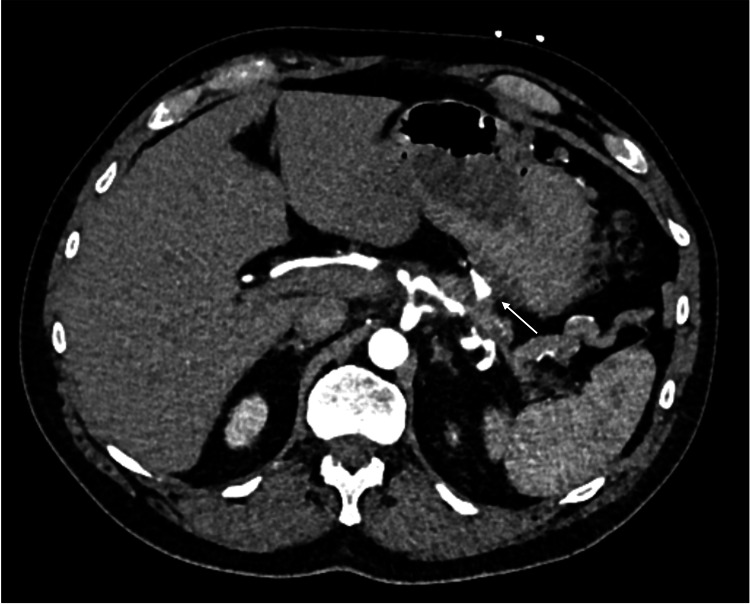
CT angiogram (transverse plane) showing left gastric artery territory pseudoaneurysm

**Figure 2:**
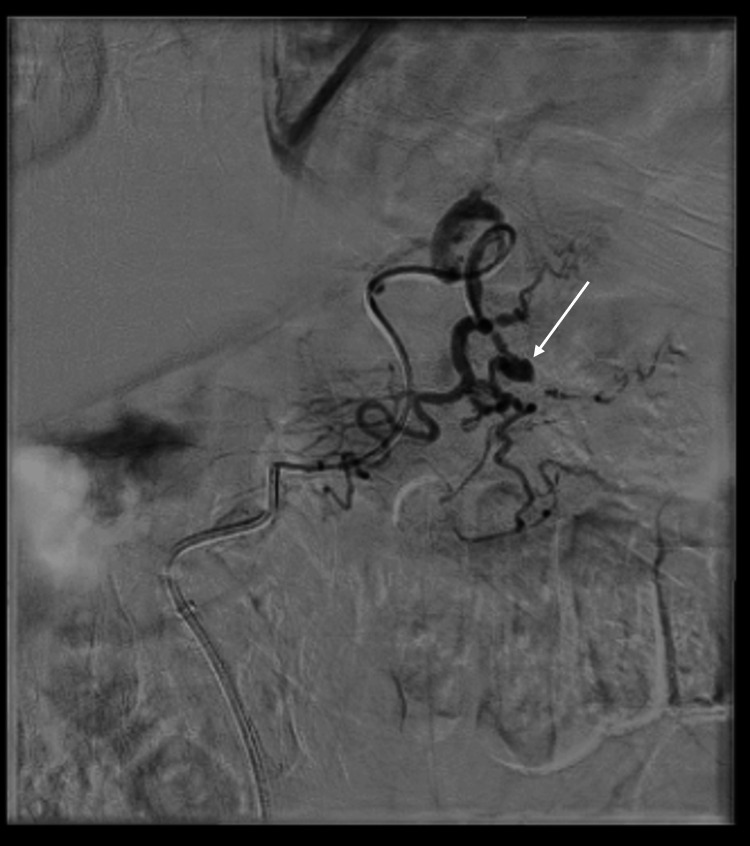
Left gastric artery pseudoaneurysm

**Figure 3:**
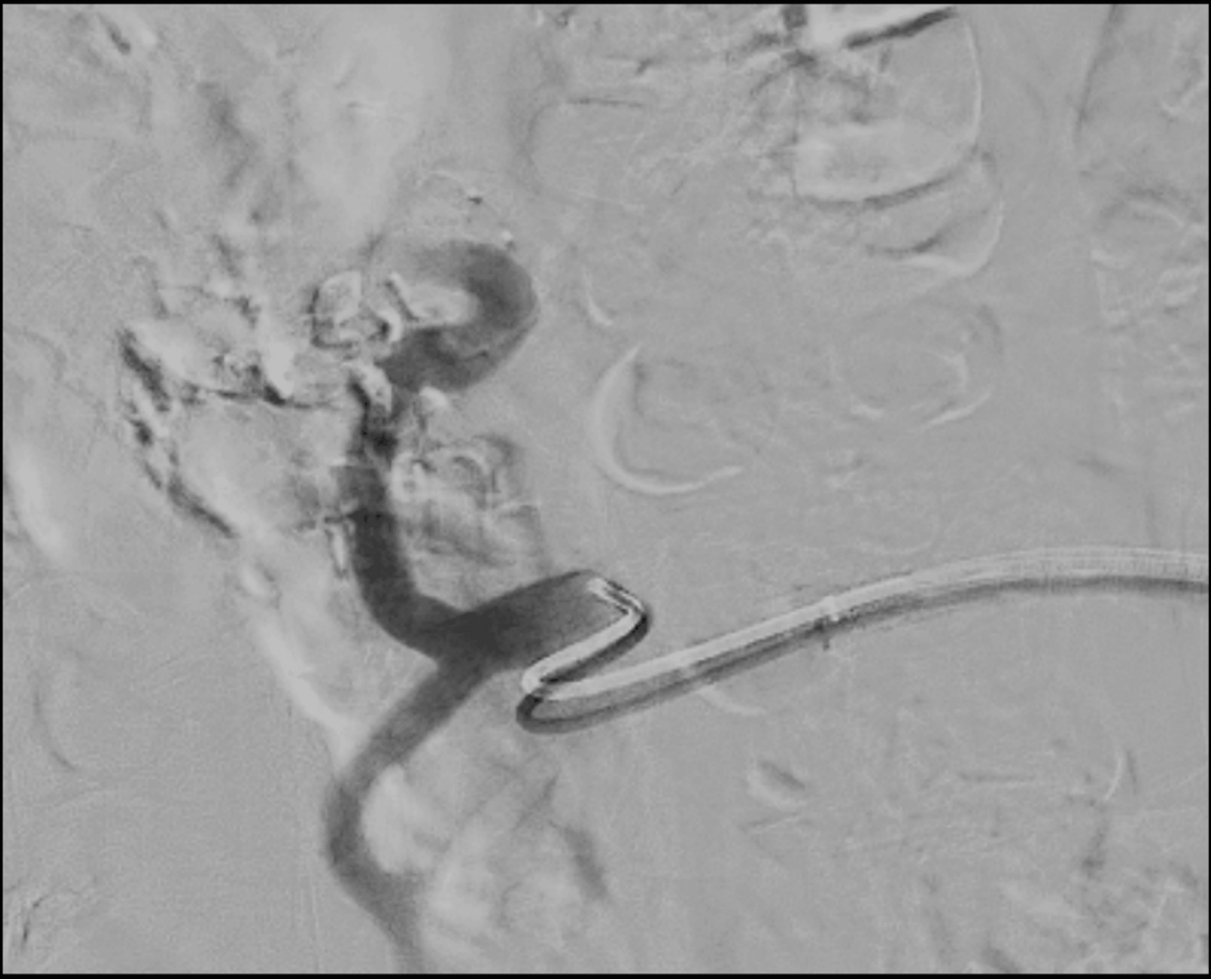
Post left gastric artery embolisation

CTA confirmed chronic splenic vein thrombosis with extensive upper abdominal varices and identified a new pseudoaneurysm in the territory of the left gastric artery (Figure 1). This pseudoaneurysm was considered the probable cause of his anaemia, and the patient underwent interventional radiology (IR) embolisation (Figures 2, 3).

**Figure 4:**
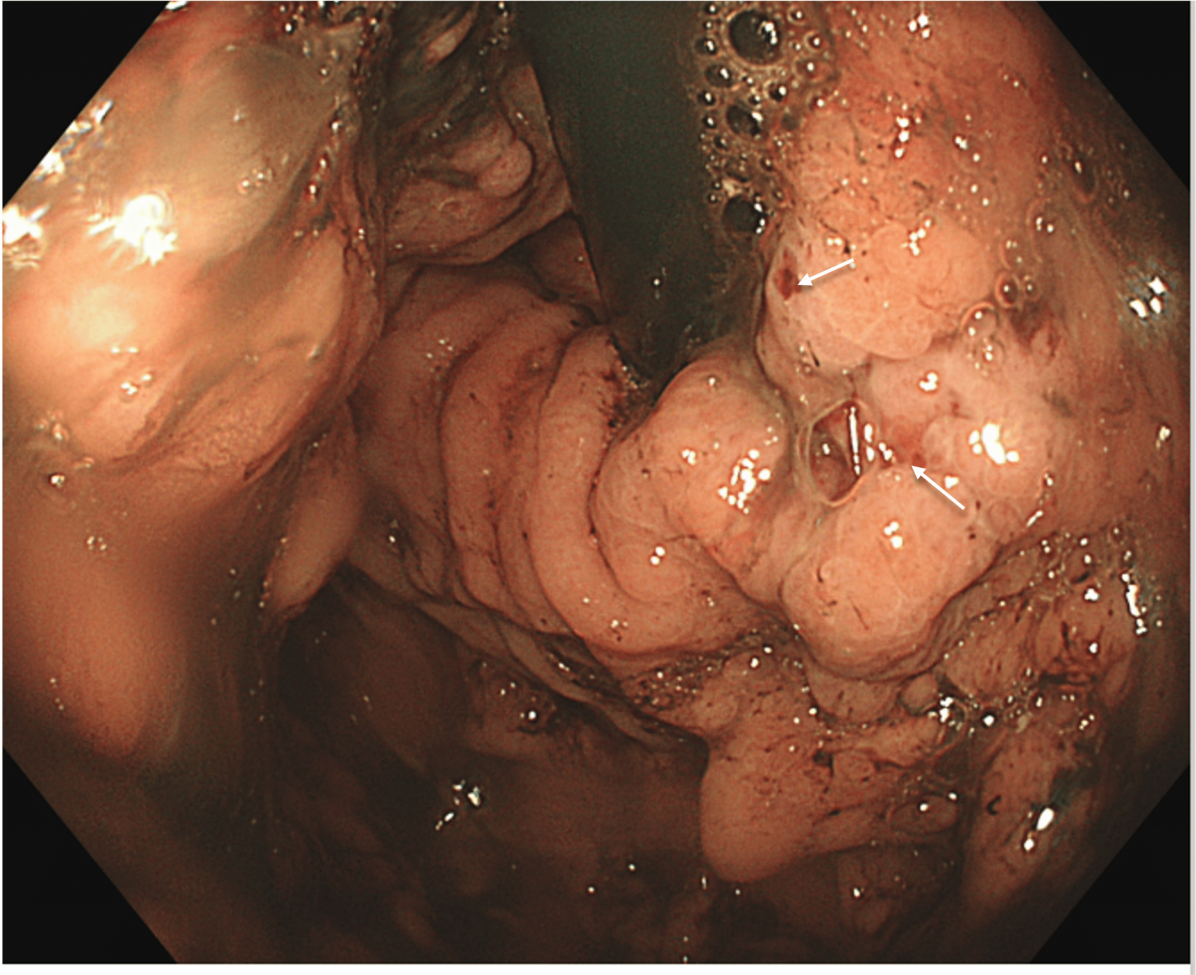
OGD day 1 post embolisation showing active gastric variceal bleeding; white arrows post to areas of possible recent bleeding OGD: oesophagogastroduodenoscopy

On the first day post-embolisation, the patient developed an episode of hematemesis. Although his haemoglobin levels initially remained stable, an OGD was performed, revealing two large gastric varices (GOV) with signs of recent bleeding (Figure 4). One varix was successfully treated with glue, and the patient received five units of blood following the procedure.

**Figure 5:**
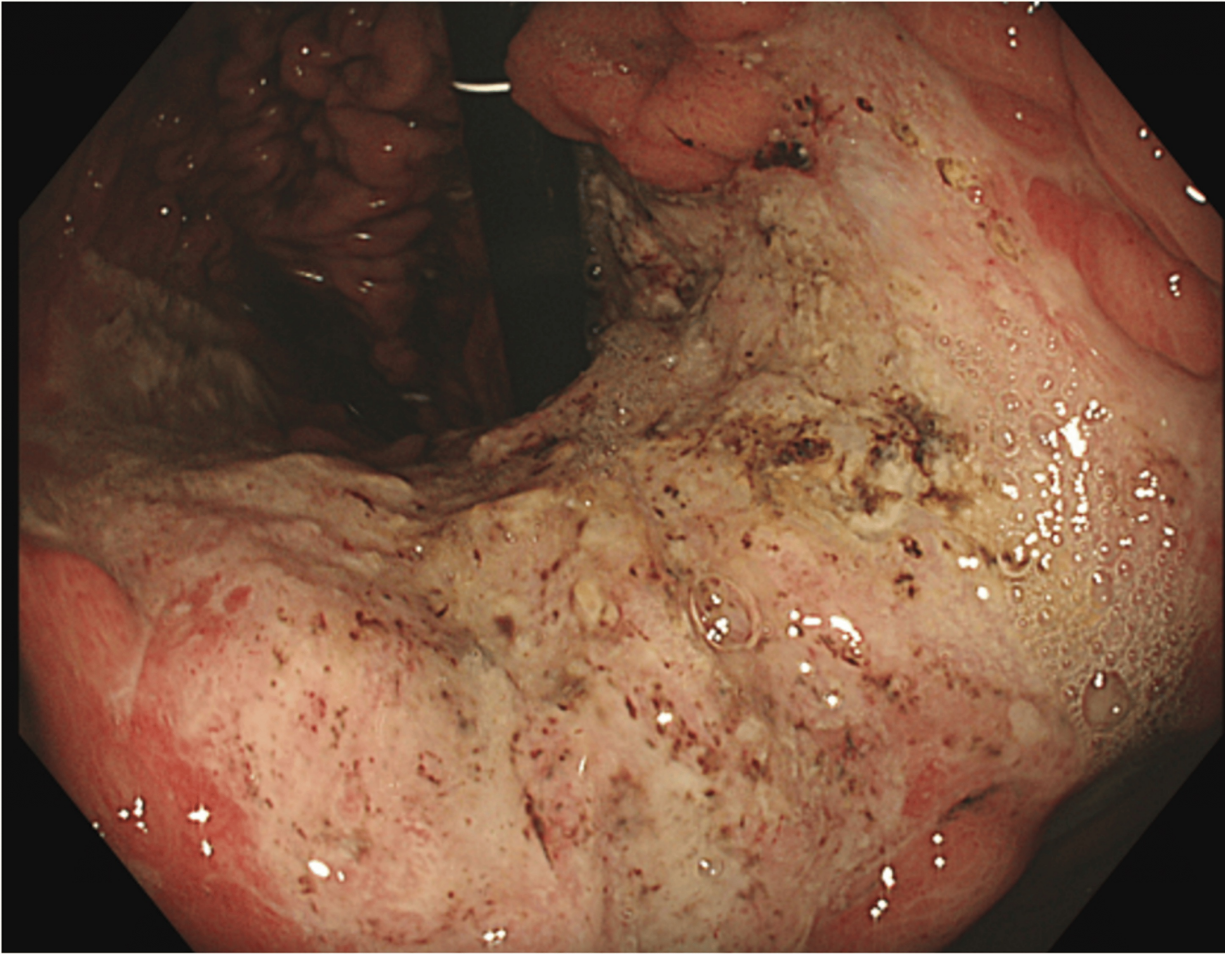
OGD day 7 post-embolisation showing a large, clean-based ulcer in the lesser curvature OGD: oesophagogastroduodenoscopy

A repeat OGD performed one week later revealed a large clean-based ulcer in the lesser curvature of the stomach (Figure 5), with no further evidence of gastric variceal bleeding. The patient was subsequently referred for consideration of splenectomy to manage his varices.

## Discussion

Following embolisation of the left gastric artery pseudoaneurysm, we propose anatomical and physiological mechanisms that may have contributed to the subsequent variceal bleeding. Anatomically, the left gastric artery, splenic artery (SA), and common hepatic artery (CHA) originate from the celiac trunk. Embolisation of the left gastric artery could have caused a redistribution of blood flow to the SA and CHA, thereby increasing pressure within the splenic vein. This phenomenon is the reverse of the therapeutic approach used to treat gastric varices secondary to splenic vein thrombosis, where splenic artery embolisation is employed to reduce blood flow [4-5].

In this patient, varices were secondary to increased splenic vein thrombosis, and the redistribution of blood flow may have exacerbated the elevated venous pressure, contributing to the bleeding. A similar case report described oesophageal variceal bleeding following lower splenic pulp embolisation, attributing it to increased blood flow in the splenogastric veins [6].

Additionally, local ischemia and tissue stress may have triggered the release of vasoactive substances, such as nitric oxide (NO), which is known to cause splenic vasodilation and increased blood flow within the portal system [7]. This rise in portal pressure likely contributed to the variceal bleeding, as evidenced by the large ischemic ulcer observed in the left gastric artery distribution during the repeat OGD.

## Conclusions

Patients undergoing interventional radiology procedures for UGIB in the context of portal hypertension are rare but present complex clinical challenges. Clinicians managing these patients should consider early OGD after embolisation to assess for potential variceal bleeding. Additionally, elective embolisation of the left gastric artery should be approached with caution in patients with underlying portal hypertension to mitigate the risk of exacerbating variceal pressures.
